# Tactile versus motor imagery: differences in corticospinal excitability assessed with single-pulse TMS

**DOI:** 10.1038/s41598-024-64665-6

**Published:** 2024-06-27

**Authors:** Marina Morozova, Aigul Nasibullina, Lev Yakovlev, Nikolay Syrov, Alexander Kaplan, Mikhail Lebedev

**Affiliations:** 1 Vladimir Zelman Center for Neurobiology and Brain Rehabilitation, Skolkovo Institute of Science and Technology, Moscow, 121205 Russia; 2 Baltic Center for Neurotechnology and Artificial Intelligence, Immanuel Kant Baltic Federal University, Kaliningrad, 236041 Russia; 3 Department of Human and Animal Physiology, Faculty of Biology, Lomonosov Moscow State University, Moscow, 119234 Russia; 4 Faculty of Mechanics and Mathematics, Lomonosov Moscow State University, Moscow, 119991 Russia; 5 Sechenov Institute of Evolutionary Physiology and Biochemistry of the Russian Academy of Sciences, Saint Petersburg, 194223 Russia

**Keywords:** Tactile imagery, Kinesthetic motor imagery, Corticospinal excitability, Transcranial magnetic stimulation (TMS), Motor evoked potentials (MEPs), Sensorimotor processing, Sensory processing, Somatosensory system

## Abstract

Tactile Imagery (TI) remains a fairly understudied phenomenon despite growing attention to this topic in recent years. Here, we investigated the effects of TI on corticospinal excitability by measuring motor evoked potentials (MEPs) induced by single-pulse transcranial magnetic stimulation (TMS). The effects of TI were compared with those of tactile stimulation (TS) and kinesthetic motor imagery (kMI). Twenty-two participants performed three tasks in randomly assigned order: imagine finger tapping (kMI); experience vibratory sensations in the middle finger (TS); and mentally reproduce the sensation of vibration (TI). MEPs increased during both kMI and TI, with a stronger increase for kMI. No statistically significant change in MEP was observed during TS. The demonstrated differential effects of kMI, TI and TS on corticospinal excitability have practical implications for devising the imagery-based and TS-based brain–computer interfaces (BCIs), particularly the ones intended to improve neurorehabilitation by evoking plasticity changes in sensorimotor circuitry.

## Introduction

Kinesthetic and tactile imagery are subdomains of the mental imagery, which is a simple but practically significant mental endeavor where actions and perceptions are imagined but not performed overtly. Most of us can imagine as simple movements as bending a finger or more complex ones such as playing the piano. This kind of motor imagery usually has both visual and kinesthetic components, that is movements are mentally visualized and also reproduced as feelings in the body parts and muscles. The latter component, which we refer to as kinesthetic motor imagery (kMI), is associated with an activation of similar neural structures as the ones that participate in the control of overt motor activity^1–3^. kMI is used in sports as a way to rehearse and improve motor performance^4^. Additionally, kMI is utilized as therapy^5^ and neurorehabilitation approach^6^.

In contrast to kMI which has been studied for decades, a somewhat different type of somatotopic imagery which we call tactile imagery (TI) has received much less attention. During TI, subjects imagine experiencing sensations arising from the skin, such as being touched with a brush or stimulated with a vibrating probe. We assert that the distinction between kMI and TI is important because kinesthetic and tactile imagery engage different types of somatosensory modalities (proprioceptive versus cutaneous) as well as incorporating imagery of various types (self-initiated movements versus passive application of stimuli). Indeed, kMI involves a mental enactment of kinesthetic sensations resulting from voluntary movements (muscle contraction, joint position, tendons tension, etc*.*) and in some versions the sense of body position^2,7–9^ whereas TI is about perceiving touch in a particular location on the skin surface^10–12^. TI specifically activates the primary somatosensory cortex^10,11,13,14^, motor-control areas^10,13^ as well as the areas generating the act of mental imagery, namely the prefrontal cortex^11^ and parietal cortex^15^. The TI-induced cortical activities can be revealed using electroencephalographic (EEG) recordings, where event-related desynchronization (ERD) of the *μ*-rhythm is the most prominent effect^12,16,17^.

While EEG-based BCIs could be considered a practical goal of the research on kMI and TI^17–19^, EEG recordings alone are insufficient for further development of such BCIs. Clearly, additional approaches are needed to study cortical processing under different imagery conditions in greater detail^20,21^. A variety of recording and stimulation methods could provide valuable insights. Here we focused on the effects of different types of imagery on corticospinal excitability assessed with the use of transcranial magnetic stimulation (TMS). Primary motor (M1) and somatosensory (S1) cortices are densely interconnected^22,23^. Corticospinal excitability, measured using TMS, is known to change during the performance of sensorimotor related tasks such as kMI and action observation^7,24–28^. Moreover, it has been shown that somatosensory inputs influence the corticospinal excitability^29,30^, making TMS an appropriate tool to study the effects of TI, as well. As to the effects on spinal excitability, kMI and TI-induced changes in the F-wave were previously reported^31^.

In the present study, our aim was to examine the influence of TI on corticospinal excitability. We hypothesized that TI would affect this excitability but differently compared to kMI because of the preferential engagement of S1 during TI. To test this hypothesis, we assessed corticospinal excitability by applying TMS pulses over M1 while participants imagined experiencing tactile sensations on the skin of the finger (TI) or, in a separate set of trials, imagined performing a finger tapping task (kMI). During the performance of these tasks, TMS was applied to the hemisphere contralateral to the examined hand, which resulted in motor-evoked potentials (MEPs) in the finger muscles. We found that MEPs differed depending on the stimulation/imagery conditions. Namely, MEPs were stronger during kMI than during TI. This finding indicates that although both types of imagery involve the somatosensory domain, they affect corticospinal excitability in different ways. The possibility of manipulating imagery to evoke differential effects on the connectivity between M1 and the spinal cord opens new directions for future research and practical applications.

## Methods

### Participants

Twenty-two healthy volunteers (4 females and 18 males, 24.5 ± 3.3 years old; mean ± standard deviation) participated in the study. All subjects self-reported as right-handed. The participants had 6–9 h of sleep prior to the TMS procedure, no alcohol or medication 24 h before the procedure, and no coffee intake in the preceding 2 h. All subjects were screened for contraindications to TMS^32^. The experimental protocol was approved by the Institutional Review Board of the Skolkovo Institute of Science and Technology and followed by the Declaration of Helsinki. All participants gave their informed consent to get involved in the study.

### Experimental design

**Figure 1:**
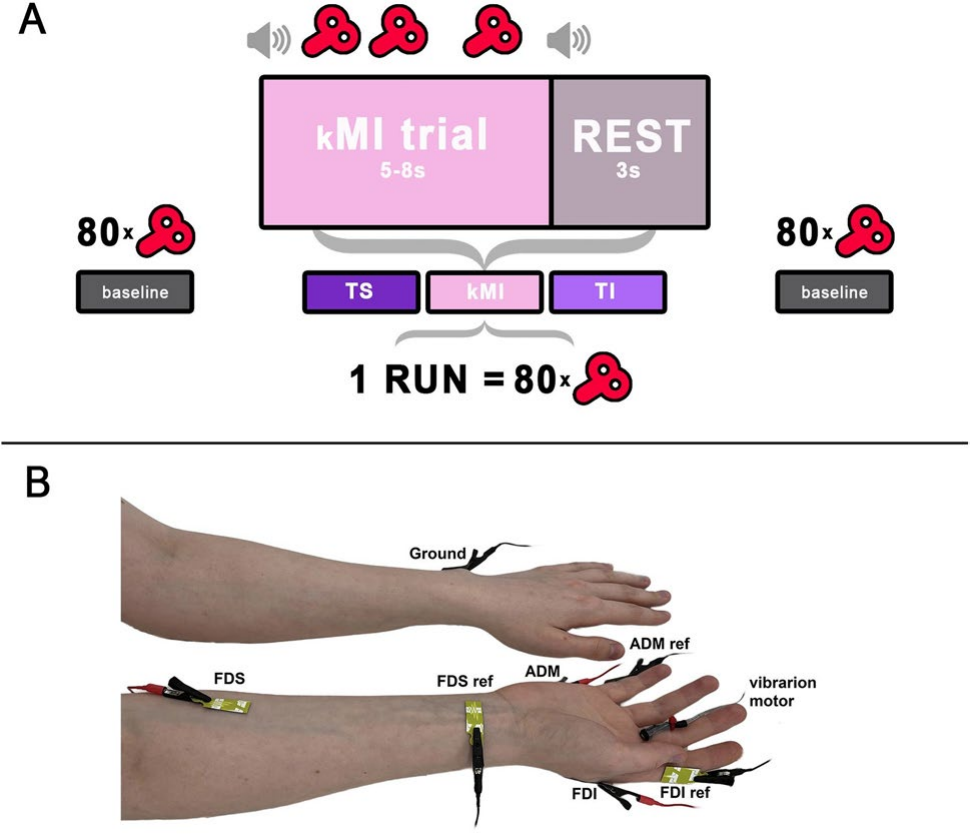
Experimental design. (**A**) Schematics of task sequence. Baseline state MEP measurements were conducted before and after the session. The task included three experimental conditions (TS, kMI and TI) performed in separate runs. Each run consisted of several trials lasting 5–8 s of a period of task performance followed by a 3-s period of resting. The total number of TMS pulses was 80 for each condition. (**B**) Placement of the EMG electrodes and vibratory motor on the forearm and hand.

In the description of our experimental design (Fig. 1A), we used the recommendations of the OHBM COBIDAS MEEG committee for reproducible EEG and MEG research^33^. Each participant participated in one experimental session lasting approximately 1.5–2 h. The preparatory procedures included detailed explanations, instructions, and the completion of informed consent forms.

Following the preparatory procedures, the participant was seated in a comfortable TMS-chair in front of the television screen (Samsung 46″ TV, South Korea). The screen was placed at a distance of 2.5 m from the participant. Several runs corresponding to each condition (baseline, TS, TI, and kMI) were performed. Participants were asked to fix their gaze on a cross displayed on the screen during all runs.

During the baseline runs (conducted before and after the all task performance), TMS pulses were delivered to the left M1 while subjects were seated, fully relaxed, and not performing any task. The first baseline run was followed by three experimental runs (eg. TS, TI and kMI) ordered by random selection across subjects. Each of these runs consisted of consecutive trials during which the participants performed the required task. The trial duration was 5–8 s, with an intertrial interval of 3 s. This variable trial duration was implemented to reduce adaptation to the stimulation that would have been expected for prolonged vibratory stimulation at a constant frequency. Audio cues were presented to guide mental tasks. They were synced with the vibrotactile stimulator using a custom python script.

The imagery trials began with an audio-verbal cue «imagine». The TS trials began with the audio-verbal cue «feel». The task performance on the trials was terminated with a beep instructing participants to stop performing the task and rest. During each trial, 2–3 randomly timed TMS pulses were applied by the experimenter manually to the left M1 with an interpulse interval of not less than 2 s. EMG activity was monitored during each trial to control for muscle activity and avoid delivering TMS during the periods of increased EMG activity. EMG recordings were synced with the TMS device using the built-in Nexstim NBS software (Nextim, Finland).

Each run ended after a total of 80 TMS pulses were applied. Due to these settings, the number of trials per condition varied from 30 to 40 in order to collect a consistent number of MEPs for each condition. After both imagery tasks and TS were completed, the session was concluded with the second baseline run.

Before the TI run, the participants were acquainted with the tactile sensations produced by vibration of proximal parts of the index, middle, ring and little fingers. Additionally, we evoked tactile sensations with a brush of these fingers and the palm and gave the opportunity to practice TI in different ways (e.g. imagery brushing at different locations of palm and fingers). The brush was used to acquaint subjects with a variety of tactile sensations. During the TI run, participants were asked to mentally reproduce the tactile sensations as vividly as if they were emerging from the skin of their fingers^12,16^. The participants were instructed not to look at the hand and instead direct their gaze to the fixation cross. During the TS task, participants were instructed to attend to skin sensations at the base of the middle finger produced with a coin-sized vibrating motor. During the kMI task, the participants mentally reproduced the kinesthetic sensations associated with performing finger movements. The finger movements were flexions and extensions at the metacarpophalangeal joint—the action often called finger tapping. We chose this specific type of movement based on our previous work, where participants learned it with ease and comfortably performed it repetitively^20,21^. Prior to the kMI run, the participants practiced performing finger movements at various speeds and executing isometric contractions and memorized the kinesthetic sensations associated with the movements, including muscle contraction, tendon tension, and joint position. The imagined finger movements were to be performed in a random order^21^.

### EMG

Surface EMG recordings were conducted using bipolar probes placed over the right *m. flexor digitorum superficialis* (FDS), *m. first dorsal interosseous* (FDI) and *m. abductor digiti minimi* (ADM). These specific muscles were chosen because they were the ones that enact the finger movements imagined during the kMI task. During the TI, the skin of the same fingers was imagined being vibrated. The active electrode was placed over the muscle belly, the reference electrode over the tendon distally, and the ground electrode was placed on the wrist of the opposite (left) hand (Fig. 1B). EMG data were sampled at 3 kHz using the built-in Nexstim EMG system (Nexstim Ltd., Helsinki, Finland).

### TMS

Single-pulse, biphasic TMS pulses were applied to the left primary motor cortex (M1) using a cooling co-planar figure-of-eight induction coil (d = 70 mm; Nextim, Finland) connected to an eXimia magnetic stimulator (Nexstim, Finland, v3.2.2). The resting motor threshold (RMT) for the FDS muscle was determined at the beginning of experimental session using the Nexstim's built-in threshold detection system as the minimum stimulation intensity (% of maximum stimulation output) required to elicit peak-to-peak MEP amplitudes in the FDS muscle that exceeded 50 μV in 5 out of 10 consecutive pulses^34,35^. During the experimental session, the stimulation intensity was 110% of the RMT. The coil orientation was kept constant using the eXimia NBS navigation system (Nexstim, Finland). Coil tracker accuracy (3D rms error) was ± 2 mm for the position and ± 2° for the angle.

### Vibrotactile stimulation

TS was delivered using a custom designed vibrotactile stimulator based on Arduino UNO (Arduino, Italy) synced with stimulus presentation software. A flat vibratory motor (d = 6 mm; max speed of 12000 rpm) was placed on the middle finger of the right hand. Variable-frequency stimulation patterns were used: stimulation was delivered as 100-ms pulses with vibratory frequency selected in the range 6000–12000 rpm, with the inter-pulse intervals varying in the range 200–400 ms. This random stimulation pattern was used to reduce tactile habituation and residual tactile sensations^12,16^.

### Data analysis

Data preprocessing and analysis was performed using Python data processing packages, including MNE 1.3.1, SciPy 1.11.1, Pingouin 0.5.4 and Statsmodels 0.14.1.

The 5 Hz high-pass filter and 50 Hz notch filter were applied to the raw EMG recordings to remove slow drifts and power line noise. The data analysis started with splitting the EMG records into the epochs timed − 0.5 to 0.1 s relative to the occurrence of the TMS pulse. These epochs were then baseline corrected by subtracting the mean for the − 0.5 to 0 s window. The epochs with EMG RMS greater than 10 μV for the − 0.5 to 0 s window were excluded from the subsequent analysis (4.8 ± 7.0% epochs across participants). Next, we calculated the peak-to-peak EMG amplitude by computing the difference between the maximum and minimum values within the 10 to 50 ms temporal window relative to the stimuli onset. For each participant, individual values of first (Q1) and third (Q3) quartiles as well as interquartile range (IQR) for FDS peak-to-peak amplitudes were calculated. The epochs with FDS peak-to-peak amplitudes that are more than 1.5 IQR below Q1 or more than 1.5 IQR above Q3 were considered outliers and removed from the subsequent analysis (4.1 ± 3.2% epochs across participants). We used FDS to determine outliers since FDS muscle hotspot was used for stimulation throughout the experiment. We then calculated the mean MEP peak-to-peak amplitude for each participant and each condition (the baselines, kMI, TI, TS).

To account for the inter-individual variability, we percent normalized the mean peak-to-peak amplitude for the kMI, TI and TS conditions to the baseline MEPs collected at the beginning of the experimental session:

1 $$\frac{{A_{cond} - A_{base} }}{{A_{base} }} \cdot 100\%$$

**Figure 2:**
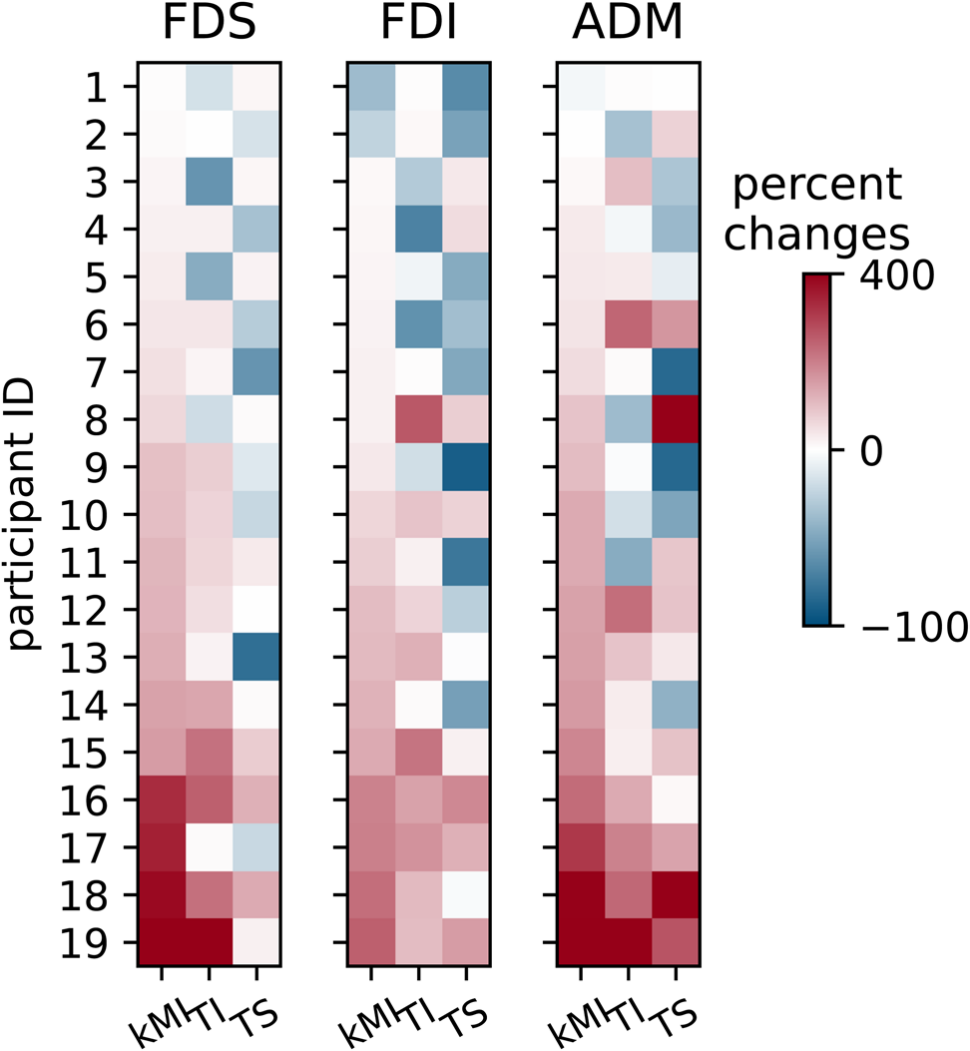
Mean MEPs for each of 19 participants (color coded). The values represent changes (as percentage) from the baseline preceding the task performance. Data are shown for the kMI, TI and TS conditions and for 3 muscles: FDS, FDI and ADM.

Positive values of this metric corresponded to a MEP increase from the baseline and negative corresponded to a decrease (Fig. 2). The metric was calculated for each participant, and then statistical analyses were conducted for the sample of participants.

The Shapiro–Wilk test of normality was used to determine whether the MEP amplitude values for each muscle were normally distributed. The normality assumption was violated for the FDS muscle in the MI (W = 0.74, p = 0.0002) and TI (W = 0.64, p = 0.000013) conditions, and for the ADM muscle in the MI (W = 0.85, p = 0.00084), TI (W = 0.83, p = 0.0035), and TS (W = 0.78, p = 0.00064) conditions. The normality assumption was also violated for the baseline runs for the FDS (W = 0.81, p = 0.0018), FDI (W = 0.85, p = 0.0061), and ADM (W = 0.771, p = 0.00044) muscles. Additionally, the normality assumption was violated for the baseline preceding the experimental conditions for the ADM muscle (W = 0.882, p = 0.023).

Due to the violations of normality, the non-parametric Friedman test and the subsequent Wilcoxon signed-rank test for pairwise comparisons were performed. This approach was chosen to maintain the validity of statistical inference for potentially non-normal distributions and small sample sizes.

Pairwise comparisons were conducted to determine statistically significant differences across the MI, TI, and TS conditions and to assess whether there were differences in the baselines before and after all task performances. A one-sample Wilcoxon test was used to statistically compare the normalized mean peak-to-peak amplitudes in the three experimental conditions against zero. This test determined whether there were significant changes in the MEP amplitude compared to the baseline. The Benjamini–Hochberg FDR correction^36^ for multiple comparisons was applied to the resulting p-values. To assess the across-participant correlations between the MEP amplitudes in different conditions, Spearman’s rank correlation coefficient was used.

## Results

Three participants were excluded from the analysis because they reported being unable to perform the required imagery tasks. Notably, their MEPs decreased when they attempted kMI. Because kMI is known to increase MEPs, and this increase is positively correlated with the vividness of kinesthetic motor imagery^21,37^, we reasoned that the lack of effect of kMI on MEP was equivalent to poor kMI ability, which was consistent with these subjects' self-reports.

Using a one-sample Wilcoxon test, we found that MEP amplitudes in the MI and TI conditions were significantly greater compared to the baseline level (recorded at the beginning of the experiment). This effect reached significance for each muscle: FDS (*W*_*kMI*_ = 0, *p*_*kMI*_ < 0.0001; *W*_*TI*_ = 29, *p*_*TI*_ = 0.014), FDI (*W*_*kMI*_ = 16, *p*_*kMI*_ = 0.002, *W*_*TI*_ = 37, *p*_*TI*_ = 0.03), and ADM (*W*_*kMI*_ = 2, *p*_*kMI*_ = 0.00005; *W*_*TI*_ = 38, *p*_*TI*_ = 0.031). TS did not have a significant effect on corticospinal excitability.

**Figure 3:**
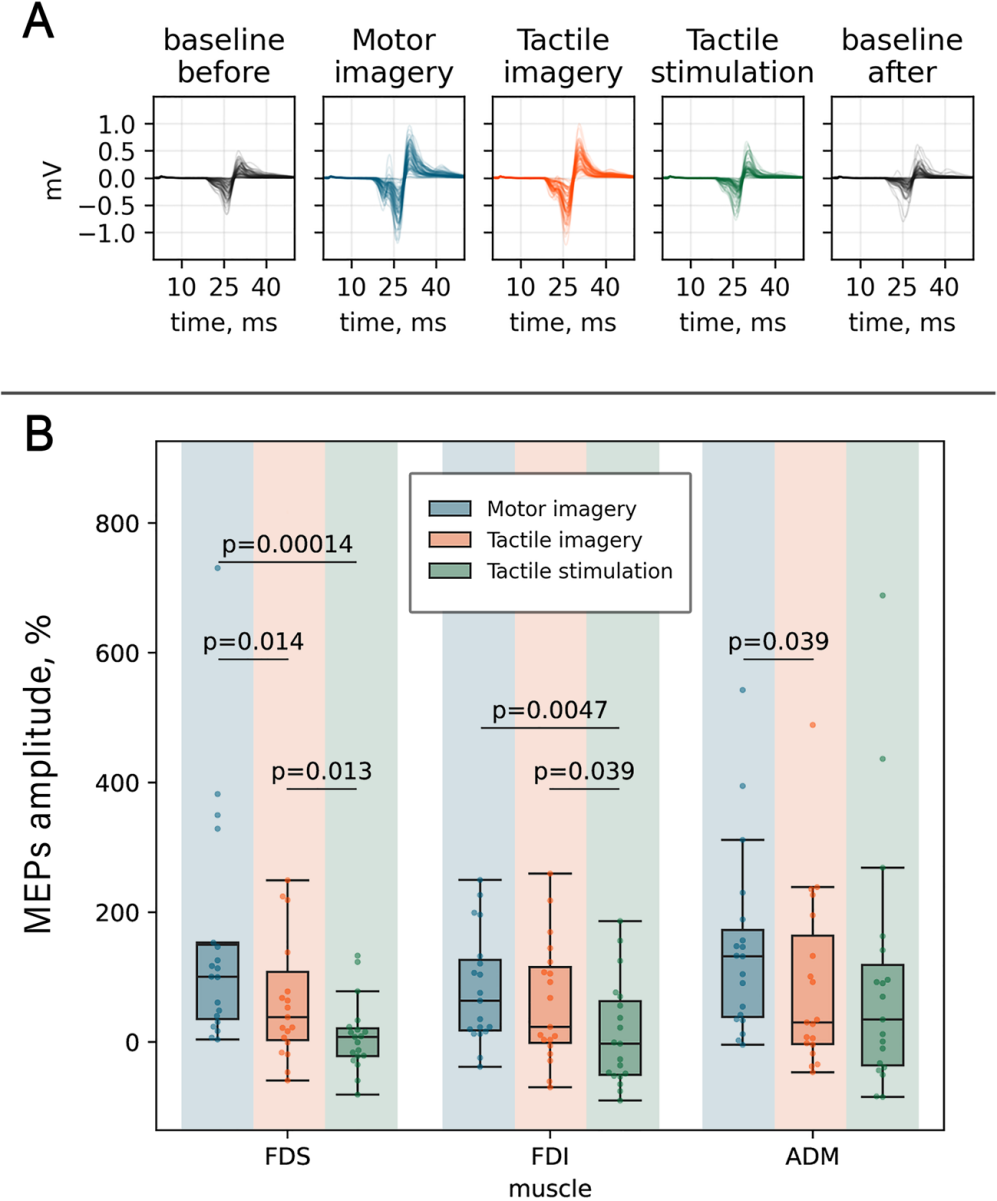
MEPs across the task conditions. (**A**) EMG epochs with MEPs for the FDS muscle in a representative participant. (**B**) Peak-to-peak normalized amplitudes across the conditions. Data are shown for three muscles. Horizontal lines within the boxes indicate median values, boxes – interquartile range (IQR) and whiskers correspond to 1.5*IQR. Dots represent single subject’ mean values. Corrected p-values are shown for the Wilcoxon signed-rank test.

Further, we compared the effects across all conditions. Friedman's test revealed statistically significant differences between kMI, TI, TS for all three muscles (Q_FDS_ = 24, p_FDS_ < 0.001; Q_FDI_ = 9.6, p_FDI_ = 0.008; Q_ADM_ = 9.6, p_ADM_ = 0.008). Therefore, the Wilcoxon signed rank test for paired samples was performed for the 9 pairwise comparisons (see Fig. 3 for results). We found that kMI resulted in a significantly greater increase in MEPs compared to the TI and TS conditions (for FDS *W*_*kMI-TI*_ = 27, *p*_*kMI-TI*_ = 0.014, *W*_*kMI-TS*_ = 2, *p*_*kMI-TS*_ = 0.00014; for FDI *W*_*kMI-TS*_ = 17, *p*_*kMI-TS*_ = 0.0047; for ADM *W*_*kMI-TI*_ = 40, *p*_*kMI-TI*_ = 0.039). MEPs recorded in TI runs were higher compared to TS, significant effects were found in FDS (*W* = 25, *p*_*TI-TS*_ = 0.013) and ADM (*W*_*TI-TS*_ = 39, *p*_*TI-TS*_ = 0.039) muscles.

Additionally, for the FDS and FDI, we found a statistically significant correlation between the MEP amplitudes during the kMI and TI conditions (Spearman’s correlation coefficient, *r*_*S, FDS*_ = 0.75, *p*_*FDS*_ = 0.0002; *r*_*S, FDI*_ = 0.7, *p*_*FDS*_ = 0.001).

**Figure 4:**
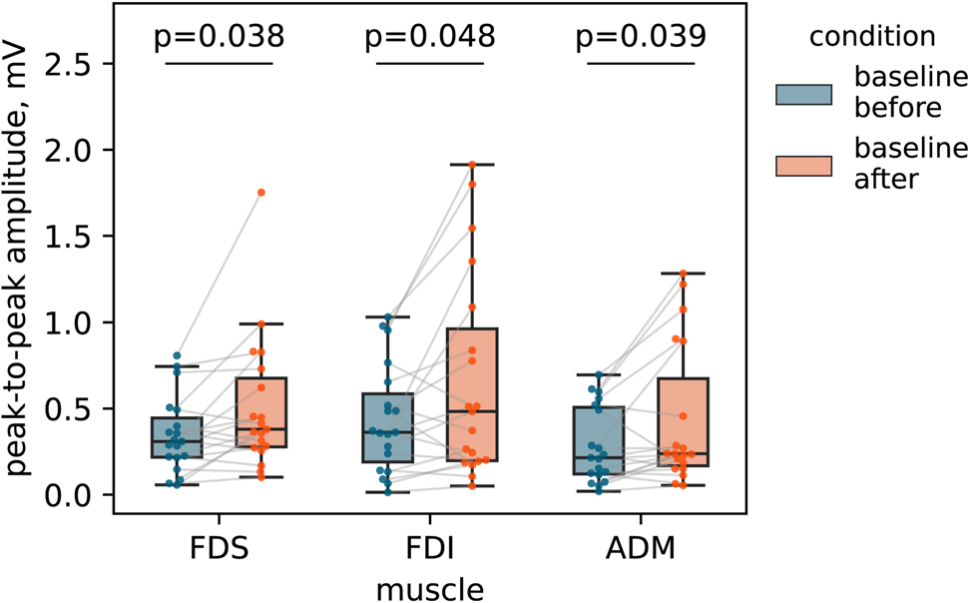
Peak-to-peak MEP amplitudes for the initial and last baselines for three muscles (*m. flexor digitorum superficialis*, FDS; *m. first dorsal interosseous*, FDI; *m. abductor digiti minimi*, ADM).  Horizontal lines within the boxes indicate median values, boxes – interquartile range (IQR) and whiskers correspond to 1.5*IQR. Dots represent single subject’ mean values. Corrected p-values are shown for the Wilcoxon signed-rank test.

To determine whether the sensorimotor tasks performed by participants during the session affected baseline motor excitability, we compared MEP amplitudes from the first and last baseline runs. The baseline run after all conditions showed significantly increased MEP amplitudes for all three muscles, namely, FDS (*W* = 36, *p* = 0.038), FDI (*W* = 43, *p* = 0.048), ADM (*W* = 40, *p* = 0.039) (Fig. 4).

## Discussion

The current study adds to the previous reports of the EEG sensorimotor rhythms being modulated by TI^12,16,17^. We explored in greater detail the differences in effects that TI, kMI and TS exhibited on the TMS-evoked responses in the finger muscles. In agreement with the numerous previous studies^7,26,27,38^, which support the interpretation that M1 is involved in MI. As such, the kMI condition could serve as a reference to which the effects of TS and TI could be compared. Yet the comparison of the effects of kMI and TI revealed that TI had a weaker facilitating effect on excitability, the TI task still demonstrated increased MEPs compared to baseline. Though this result might seem unexpectable, the observed tendency for both types of imagery to affect corticospinal excitability aligns with the findings of the previous study on measuring spinal motor neuron excitability by analyzing the F-wave^31^. Similar to our findings, that study demonstrated that while both kMI and somatosensory imagery increased F-wave persistence, the effects of kMI were stronger^31^. Along with the possible effects of somatosensory imagery on spinal excitability, Bunno discusses the possibility of unintentional muscle contraction during TI. This was observed in their study where participants imagined tactile perception while holding a sensor (pinch meter) between the thumb and index finger. However, in our study, participants performed all tasks in a resting state, which was also confirmed by EMG monitoring. Therefore, such an argument does not support our results' explanation. To mitigate the likelihood of inaccurate imagery strategies (e.g., focusing on muscles during the imagery), we administered brush stimulation to the fingers prior to the TI runs, primarily targeting tactile skin receptors. Therefore, while we cannot entirely rule out the involvement of kinesthetic sensation in individual imagery strategies, we consider its impact to be minor. According to the neuroimaging studies, TI may engage the precentral non-primary motor regions (premotor cortex, supplementary motor area^10,11,13,39^) in addition to the postcentral areas. These regions have direct projections to both M1 cortex and the spinal cord^40–42^, potentially affecting corticospinal excitability. Moreover, these corticospinal projections may be involved in the recovery mechanisms that occur during M1 impairment^43–45^. These projections could explain some of the effects of TI. Given the previous results^31^, the increase of the MEP amplitudes we observed during TI could have originated in the spinal circuitry. Another possible explanation for the increased MEP amplitudes during TI is that the participants focused their attention on the hand during both kMI and TI^11,46^. Such attention to body parts was reported to influence corticospinal excitability^47–49^.

We observed a strong across-subject correlation between the MEP amplitudes during kMI and TI, which could have reflected individual differences in the abilities to process mental images. This correlation is explainable by the involvement of the similar attentional mechanisms when focusing on the imagined hand. In our research, we primarily focused on the distinct effects of different imagery tasks (kMI, TI) on MEP amplitudes. It is also important to note that incorporating specific psychometric questionnaires, such as the Kinesthetic and Visual Motor Imagery Questionnaire (KVIQ)^50^ and Tactile Imagery Vividness Questionnaire (TIVQ)^51^, could provide a more comprehensive assessment of the individual participants' abilities. These questionnaires could help evaluate the strength and vividness of mental imagery in the somatosensory domain.

Interestingly, while we found TI facilitation of corticospinal excitability, our study did not reveal significant effects of TS on MEP amplitudes. The pertinent literature provides conflicting evidence regarding the possible effects of vibrotactile stimulation on cortical and spinal cord activity^52^. Namely, the effects of vibration depend on many factors, such as frequency, amplitude, exposure period and location of the stimulating probe^52–56^.

In our study, we applied short vibration trains (4–6 s) consisting of 100 ms bursts with varying vibration frequency (measured in rpm: 6000–12000, corresponding to 100–150 Hz) and interstimulus intervals (200–400 ms, corresponding to a burst frequency of 2–3.33 Hz). We employed a variable stimulation pattern to prevent receptor accommodation and to ensure participants’ sustained attention to the stimuli. However, this complexity also made it difficult to isolate specific effects of vibrotactile stimulation on corticospinal excitability. In the literature, vibrotactile stimulation is often described as focal muscle vibration (FMV) aimed at activating muscle spindles. In contrast to these studies, our conditions were adjusted to target only the skin surface. First, the amplitude of vibration was low in our study, and the rotor oscillations occurred in the horizontal axis relative to the skin surface, which did not apply pressure and stretch to the tissue and reduced muscle spindle activation. We did not observe any motor responses to vibration such as tonic vibration reflex, and the subjects did not report any illusory sensation of movement, which would be a sign of a strong muscle spindle activation by FMV^56,57^. Second, most studies using FMV for cortical modulation use prolonged stimulation periods (10–30 min) with constant frequency^52–55^, whereas our study had varied stimulation frequencies and intervals. In conclusion, although we cannot exclude the possibility of tactile-kinesthetic effects during TS, the primary effect of the vibration used was on tactile sensations.

Finally, we observed a moderate increase in the baseline MEP amplitudes recorded at the end of the experiment compared to the baseline MEP amplitudes recorded at the beginning (Fig. 4). In explaining this result, we propose several hypotheses. First, it is well-documented that training involving kMI leads to a sustained increase in motor cortex excitability^58^, it is plausible that both kMI and the remaining tasks could have contributed to the observed increase in baseline excitability. Concurrently, the observed increase in MEP amplitudes could be attributed to a cumulative effect induced by the sequence of single TMS pulse^59^. Notably, since the kMI, TI, and TS conditions were randomly ordered across participants, this phenomenon cannot account for the observed effects of these experimental conditions.

Despite the clear evidence provided by the results of the current study that different imagery strategies lead to distinct changes in corticospinal excitability, as measured by single-pulse TMS applied over the M1, it is important to acknowledge several limitations of our work.

First and foremost, the effects of the vibratory stimulation utilized in this work are not limited to the activation of cutaneous receptors. Such vibration may activate muscle receptors, as well, so it is challenging to produce purely tactile perceptions with such stimulus. To mitigate this disadvantage, alternative stimulation methods, including brush stimulation, von Frey filaments, and pressure-based tactile stimulators, could be employed.

Secondly, we assessed corticospinal excitability by measuring MEPs produced with the TMS coil placed over M1. This stimulation protocol could be enriched by other stimulation paradigms such as TMS-EEG recordings, paired-pulse TMS, cervical magnetic stimulation and H-reflex and F-wave testing. These additional methods could contribute to more robust conclusions regarding the effects of TI on corticospinal excitability.

An additional limitation of our study is the absence of psychological assessments, which could have provided valuable insights into the individual mental imagery capacities. Future work should consider integrating these questionnaires to gain a deeper understanding of the underlying mechanisms related to mental imagery. Implementing such assessments in future studies can offer more nuanced insights into the variability of imagery skills and their impact on corticospinal excitability. The use of these questionnaires should be implemented in future work, as they can provide more relevant insights into the mechanisms underlying the strength and vividness of mental imagery in the somatosensory domain.

Overall, our study has demonstrated that single-pulse TMS of M1 is specific enough to dissociate the effects of TI and kMI on corticospinal excitability. This finding contributes to our understanding of the mechanisms underlying mental imagery, especially in the context of sensorimotor integration. It also paves the way to the development of the imagery-based BCI applications, where kMI and TI exert different effects on the activity of sensorimotor circuits. Such BCI implementations could be used for monitoring and manipulating corticospinal excitability in order to facilitate the rehabilitation of sensorimotor functions in patients suffering from the consequences of stroke and other neural disorders. Distinguishing between different types of somatosensory mental imagery can help to guide the rehabilitation procedures more precisely by accounting for the specifics of brain damage and the affected sensorimotor functions.

## Data Availability

The dataset collected and analyzed during the current study is available from the corresponding author on reasonable request.
